# Analysis of size and shape differences between ancient and present-day Italian crania using metrics and geometric morphometrics based on multislice computed tomography

**DOI:** 10.1080/20961790.2017.1338041

**Published:** 2017-06-20

**Authors:** Fabrice Dedouit, Giuseppe Guglielmi, Astrid Olier, Frédéric Savall, Michelangelo Nasuto, Theodorus Thanassoulas, Roberto Grassi, Alfonso Reginelli, Salvatore Cappabianca, Norbert Telmon

**Affiliations:** a Unit of Forensic and Anthropological Imaging, University Center of Legal Medicine Lausanne-Geneva, Lausanne, Switzerland; b Laboratory of Biological Anthropology AMIS, UMR 5288 CNRS, Université Paul Sabatier, Toulouse, France; c Department of Radiology, University of Foggia, Foggia, Italy; d Department of Radiology, Scientific Institute Hospital “Casa Sollievo della Sofferenza”, San Giovanni Rotondo, Italy; e Institute of Legal Medicine, CHU Toulouse-Rangueil, Toulouse Cedex 9, France; f Human Anatomy Unit, Department of Public, Clinical and Preventive Medicine, Second University of Naples, Naples, Italy; g Unit of Radiology, Radiotherapy and Nuclear Medicine, Department of Experimental and Clinical Internistic “F.Magrassi, A.Lanzara”, Second University of Naples, Naples, Italy

**Keywords:** Forensic science, forensic anthropology, craniometry, computed tomography, landmarks, morphometry, geometric, Italian crania

## Abstract

The Museum of Human Anatomy in Naples houses a collection of ancient Graeco-Roman crania. The aim of this study was to use multislice computed tomography (MSCT) to evaluate and objectively quantify potential differences in cranial dimensions and shapes between ancient Graeco-Roman crania (*n* = 36) and modern-day southern Italian crania (*n* = 35) and then to characterize the cranial changes occurring over more than 2000 years, known as secular change. The authors used traditional metric criteria and morphometric geometry to compare shape differences between the sets of crania. Statistically significant differences in size between the ancient and modern crania included shorter facial length, narrower external palate, smaller minimum cranial breadth, shorter right and left mastoid processes, and wider maximum occipital and nasal breadth. The shape changes from the ancient to modern crania included a global coronal enlargement of the face and cranial diameters, with more anterior projection of the face at the anterior nasal spine, but also posterior projection at the glabella and the nasion. It is not possible to determine whether these differences result exclusively from secular changes in the cranium or from other factors, including a mix of secular change and other unknown factors. To the best of our knowledge, this is the first MSCT-based study to compare ancient Graeco-Roman and modern-day southern Italian crania and to characterize shape and size differences.

## Introduction

The Museum of Human Anatomy at the Second University of Naples houses unique collections of anatomical specimens, including a series of ancient crania found in the necropolis of Pontecagnano and Pompeii in the region of Campania in southern Italy [1]. This series was dated between the seventh and fifth centuries BC and defined as ancient Graeco-Roman crania. Out of this collection, 36 crania without their mandibular bones and in varying states of preservation were studied with multislice computed tomography (MSCT). The authors had no information concerning the subjects before death, such as social status, sex, or age at death, except that they were all mature individuals.

The aim of this study was to evaluate and objectively quantify potential variations over time with the novel tool of MSCT. The authors used morphometric geometry to compare shape and angle differences between ancient and modern crania. The study focused on cranial dimensions and shape differences between an ancient Graeco-Roman sample and a modern-day sample from southern Italy, aiming to describe cranial changes over a 2000-year period known as secular change.

## Materials and methods

This work exclusively focused on potential craniometric differences between ancient Graeco-Roman and modern-day southern Italian subjects. Anthropological criteria including estimation of sex and age at death were evaluated. Because the ancient Graeco-Roman crania were archaeological remains, the authors chose not to apply the craniometric method of sex determination developed by Giles and Elliot because that method was derived and calculated exclusively from modern crania [2]. Furthermore, given the different states of conservation of the ancient crania, application of the formulae described by Giles and Elliot would result in a theoretical percentage of correct sex determination ranging from 84.5% to 86.6% based on percentages given in the original publication [2]. Consequently, because of the small sample size and the potential for false sex attribution, we decided to divide the sample into two groups, which we called ancient Graeco-Roman crania and modern crania, without consideration of subject sex.

### Materials

#### Crania

The ancient Graeco-Roman crania series was dated between the seventh and fifth centuries BC and was defined as ancient Graeco-Roman crania. Some of the crania were donated to the Museum from the excavations at Pompeii, Herculaneum, Cumae, and Teste della Vicaria (southern Italy) by Prof. Gennaro Barbarisi, director of the anatomy cabinet of Naples in 1870 [1]. Other crania, dating from the pre-Roman to Roman era and coming from Sarno and Pontecagnano (southern Italy), were provided by the Archaeological Committee of Salerno. Part of this collection had already been studied with MSCT [3]. Because of the conservation state of the crania, the study focused on the splanchnocranium and neurocranium. Thirty-six crania were analyzed. All were adult crania as indicated by complete fusion of the spheno-occipital synchondrosis.

The present-day sample consisted of 35 cranial scans of patients who were retrospectively included in the study. The MSCT scans performed for clinical neurological investigation were acquired at the San Giovanni de Rotondo Hospital in the Puglia region of southern Italy. These 16 women and 19 men lived in Foggia and were considered representative of the present-day southern Italian population. The images from the clinical explorations were recorded anonymously. None of the subjects had cranial bone disease or cranial deformation; the average subject age was 63.1 years.

#### Radiological features

For the ancient Graeco-Roman crania, MSCT was performed at the University of Naples, Italy (Aquilion; Toshiba Medical Systems, Tochigi, Japan). The slice thickness was 1.0 mm, with 0.5 mm collimation and a 512 × 512 matrix. Technical parameters were 100 mAs and 120 kV. Sharp kernel reconstruction was performed and images were visualized with a standardized bone window.

For the present-day subjects, the MSCT exploration was performed in the radiology department at the Hospital of San Giovanni de Rotondo, Italy (Aquilion 54; Toshiba Medical Systems, Tochigi, Japan). The slice thickness was 0.5 mm, with 0.75 mm collimation and a 512 × 521 matrix. Technical parameters were 200 mAs and 120 kV. Sharp kernel reconstruction was performed and images were visualized with a standardized bone window.

### Methods

#### Post-processing

Scans were saved as Digital Imaging and Communications in Medicine (DICOM) files and post-processing was performed with Amira 5.0.1. Software (Amira Mercury Computer Systems Solutions, Chelmsford, MA, USA). Reconstructions were two-dimensional multiplanar reformation (MPR) and three-dimensional volume rendering technique (VRT). The images from clinical explorations were recorded anonymously.

On the basis of standard anthropometric techniques and the anthropological literature, 26 craniometric landmarks were selected (Table 1). These landmarks were chosen as an adequate representation of those used in traditional craniometric methods and textbooks [4–7]. The landmarks were positioned on the MSCT reconstructions using Amira with simultaneous VRT and MPR modes. The corresponding 3D coordinates (*x*, *y*, *z*) of each landmark were subsequently recorded.

**Table 1.** Anatomical description of the 26 cranial landmarks positioned on the 3D MSCT reconstructions.

Landmark name	Abbreviation
Glabella	G
Opisthocranion	Op
Euryon Left	EuL
Euryon Right	EuR
Basion	B
Bregma	Br
Nasion	N
Zygomatic Left	ZygL
Zygomatic Right	ZygR
Prosthion	Pr
Ectomolare Left	EcmL
Ectomolare Right	EcmR
Porion Left	PoL
Process Mastoid Left	ProcMastL
Porion Right	PoR
Process Mastoid Right	ProcMastR
Frontomalare temporal Left	FtmL
Frontomalare temporal Right	FtmR
Asterion Left	AstL
Asterion Right	AstR
Alare Left	AlL
Alare Right	AlR
Frontotemporal Left	FtL
Frontotemporal Right	FtR
Sella	S
Anterior Nasal Spine	ANS

#### Data analysis

All statistical analyses and morphometric geometric analyses were performed with R 2.2.0 software (http://www.r-project.org/) [8].

##### Measurement precision

To examine the effects of intra-observer error, the principal observer carried out five observations of 10 randomized specimens from the sample of 71 crania (including ancient Graeco-Roman and modern crania) 1 month after the initial examination. To assess inter-observer error, a second observer carried out one observation of 10 randomized specimens.

##### Osteometric study

A classic osteometric study was performed first. The 26 craniometric landmarks were used to obtain 14 craniometric lengths: maximum cranial length (GOL), maximum cranial breadth (XCB), maximum cranial height (BBH), cranial base length (BNL), bizygomatic facial breadth (ZYB), facial length (BPL), upper facial length (NPH), external palate breadth (MAB), right and left mastoid process height (MDH R and MDH L), maximum transverse frontal breadth (XFB), maximum occipital breadth (ASB), nasal breadth (NBL), and minimum frontal breadth (WFB) (which are presented in Table 2).

**Table 2.** Anatomical description of the 14 craniometric measurements.

Measurements	Landmarks used	Acronyms
Maximum Cranial Lenght	Glabella–Opisthocranion	GOL
Maximum Cranial Breadth	Eurion Left–Eurion Rigth	XCB
Maximum Cranial Height	Basion–Bregma	BBH
Cranial Base Length	Basion–Nasion	BNL
Bizygomatic Facial Breadth	Zygomatic Left–Zygomatic Rigth	ZYB
Facial Length	Basion–Prosthion	BPL
Upper Facial Length	Nasion–Prosthion	NPH
External Palate Breadth	Ectomolare Left–Ectomorale Right	MAB
Mastoid Process Height (right and left)	Vertical projection of the mastoid process below and perpendicular to the Frankfurt plane	MDH R/L
Maximum Transverse Frontal Breadth	Frontomalare temporal Left–Frontomalare temporal Right	XFB
Maximum Occipital Breadth	Asterion Left–Asterion Right	ASB
Nasal Breadth	Alare Left–Alare Right	NBL
Minimum Frontal Breadth	Frontotemporale Left–Frontotemporale Right	WFB

Other useful angles and ratios were also calculated: the nasion–sella–basion (NSB), which represents the cranial base angle; the sella–nasion–anterior nasal spine (SNA), which illustrates maxillary prognathism; and neurocranial globularity, defined as (euryon–euryon × basion–bregma)/nasion–opisthocranion [3,9,10].

To assess statistically significant differences between the ancient Graeco-Roman and modern samples, Student's *t*-test was performed with calculation of the *P*-values and Bonferroni corrections. The threshold *P*- value for significance was 0.05.

##### Morphological 3D analysis: geometric morphometric analysis

A combination of landmark-based geometric morphometric analyses was used to investigate differences in cranial shape and size between the two samples. The distribution of coordinates was evaluated with a Shapiro–Wilk test to determine normality. Principal component analysis (PCA) was used as an exploratory step.

First, we carried out generalized least-squares (GLS) Procrustes superimposition of 3D landmark coordinates. This procedure quantifies shape by removing nuisance parameters such as initial differences in centroid size (CS), position, and orientation of the specimens. It defines the shape of each specimen in terms of Procrustes residuals, which serve as the starting point of the statistical analysis of shape. With generalized Procrutes analysis (GPA), size effects related to isometry were removed, but allometric size differences were retained and visible. Cranial size was represented by CS, the sum of squared distances of each landmark from the centroid (the average position of all landmarks in the configuration). CS is the only measurement size that is not correlated with shape and is therefore appropriate in GPA. CS was used in this study as a biologically meaningful expression of the overall scale of the landmark configuration, and thus of the bones studied, and made it possible to examine allometry. Allometry is the shape variation that correlates with size. In this study, static allometry was studied, corresponding to the shape variation that correlates with size.

A consensus configuration, or mean shape configuration, was produced for each sample, so that differences between inter-sample configurations could be compared. These superimposed mean antique and modern landmark configurations (consensus configuration shape) were represented three-dimensionally as graphical wireframes (with lines between landmarks). This permitted a 3D representation, individually for each group to illustrate graphically 3D deformations. Additionally, a thin-plate spline analysis was produced to illustrate 2D deformations.

A complementary analysis was performed based on extraction of the extreme CS values of both samples to objectify size differences.

## Results

### Measurement precision

Each observer calculated landmark deviations relative to the landmark mean. Percent errors were calculated for the 26 landmarks. The percentage errors for intra- and inter-observer variabilities concerning variation in landmark positioning did not exceed 3%. Previous authors have suggested that results are acceptable when the percentage errors do not exceed 5% [11–13].

### Osteometric study

The results are summarized in Table 3. There were no statistically significant differences between the groups in the following cranial dimensions (7/14): GOL, XCB, BBH, BNL, ZYB, NPH, and XFB. Additionally, there were no differences in NSB angle, SNA angle, or neurocranial globularity between the ancient Graeco-Roman and modern crania.

**Table 3.** Craniometric results.

Acronyms	A (mean±SD)	C (mean±SD)	*P*	P*
GOL(mm)	181.5 ± 8.0	180.6 ± 10.1	0.45	0.67
XCB(mm)	135.0 ± 5.5	138.1 ± 6.7	0.09	0.10
BBH(mm)	134.1 ± 4.9	131.7 ± 6.1	0.07	0.07
BNL(mm)	101.3 ± 3.7	100.8 ± 5.0	0.63	0.60
ZYB(mm)	124.3 ± 20.6	128.9 ± 5.7	0.61	0.22
**BPL**(mm)	**94.2 ± 5.4**	**89.7 ± 5.2**	**0.01**	**0.01**
NPH(mm)	59.6 ± 5.7	60.6 ± 6.1	0.58	0.59
**MAB**(mm)	**61.5 ± 3.3**	**59.0 ± 4.5**	**0.04**	**0.04**
**MDH L**(mm)	**31.6 ± 3.1**	**29.5 ± 4.1**	**0.04**	**0.02**
**MDH R**(mm)	**31.3 ± 3.5**	**29.3 ± 4.2**	**0.04**	**0.02**
XFB(mm)	91.4 ± 3.7	93.4 ± 4.6	0.14	0.14
**ASB**(mm)	**110.4 ± 6.1**	**113.9 ± 7.1**	**0.03**	**0.03**
**NBL**(mm)	**25.3 ± 1.8**	**28.0 ± 2.2**	**<0.001**	**<0.001**
**WFB**(mm)	**95.3 ± 4.1**	**93.7 ± 4.1**	**0.04**	**0.04**
Glob	0.57 ± 0.05	0.57 ± 0.05	0.72	
NSB(degrees)	137.1 ± 6.0	138.3 ± 7.0	0.30	
SNA(degrees)	82.3 ± 4.0	84.3 ± 4.3	0.18	

A, antique Greek-roman crania. C, modern crania. SD, standard deviation. Bold, significant results. Glob, neurocranial globularity defined as (euryon–euryon × basion–bregma)/nasion–opisthocranion. NSB, nasion–sella–basion which is the cranial base angle. SNA, sella–nasion–anterior nasal spine which illustrates the maxillary prognatism. *P*, *P*-value according to the Student's *t*-test. P*, *P*-value with the Bonferroni correction.

Concerning the statistically significant results (7/14), in the modern crania we noted:

- Smaller BPL, MAB, WFB, MDH R and MDH L.
- Greater ASB and NBL.

### Morphological 3D analysis: geometric morphometric analysis

**Figure 1.**
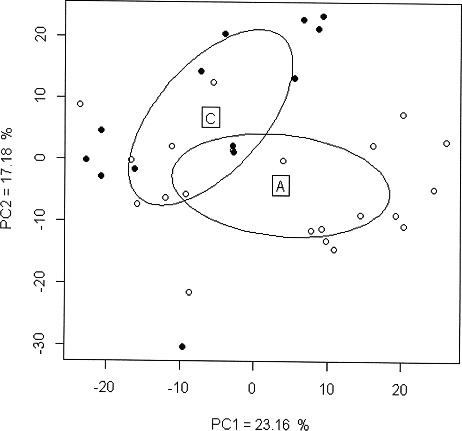
Graphical representation of a principal component analysis after generalized Procrustes analysis (GPA) based on the 3D coordinates of 24 landmarks. The principal component (PC) axes selected are those with the most significant eigenvalues (PC1–PC2). PC1 = 23.16%, PC2 = 17.18%. The ellipses represent 68% confidence intervals for ancient Graeco-Roman crania (A) and modern crania (C). White circles: ancient Graeco-Roman crania; Black circles: modern crania.

The distribution of the coordinates was determined to be normal with the Shapiro–Wilk test. The PCA obtained for cranial shape variables is presented in Figure 1. The PCA had PC1 and PC2 representing 39.75% of the explained variance, indicating that criteria other than shape differences (illustrated with the PCA) contributed to differences between the ancient and modern populations. However, the two samples were distinguishable, with a separation along the PC2.

**Figure 2.**
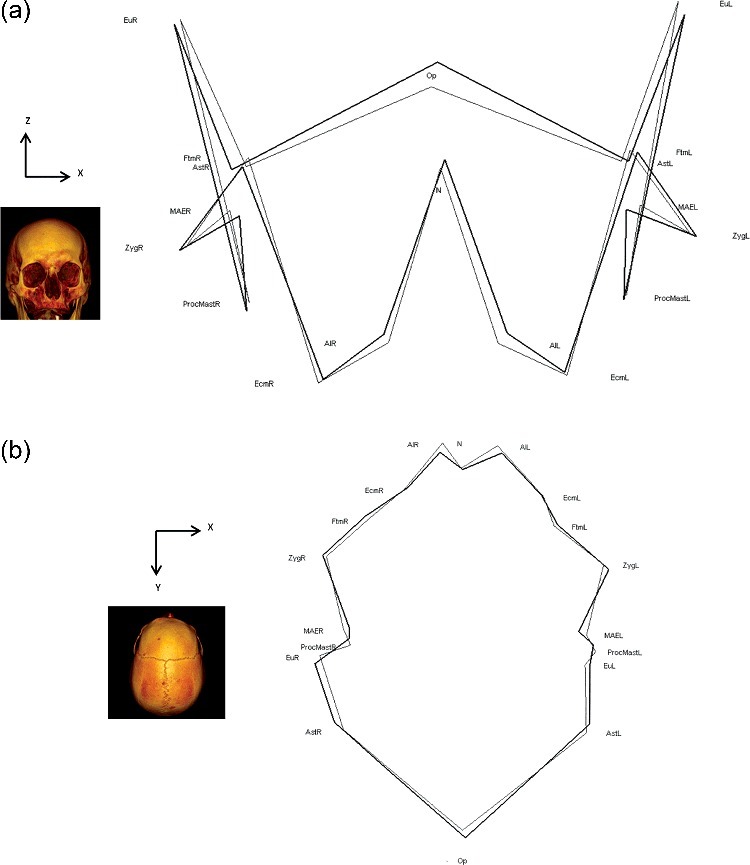
Three-dimensional (3D) graphical representations of the consensus paired landmark positions. (A) Anterior view; (B) superior view. The 3D reconstructions and the axis permit a better understanding of the orientation of the 3D graphical representation. See Table 1 for definitions of landmark abbreviations. Bold wireframes: modern crania; thin wireframes: ancient Graeco-Roman crania.

3D graphical representations concerning modifications in the paired landmarks are presented in Figure 2. For increased clarity, some landmarks are not presented (glabella, prosthion, anterior nasal spine, basion, sella, and bregma). Each landmark presented is the consensus landmark for the ancient Graeco-Roman or the modern sample, allowing for an average shape representation.

The most important shape differences and landmark displacements are:

- Posterior and external displacement of the right and left alare points (AlR and AlL). This illustrates changes in the NBL (alare left–alare right).
- External displacement of the right and left eurion, zygomatic, and asterion landmarks. This illustrates changes in the XCB (euryon left–euryon right), the ZYB (zygomatic left–zygomatic right), and ASB (asterion left–asterion right).
- Upward displacement of the opisthocranion.

**Figure 3.**
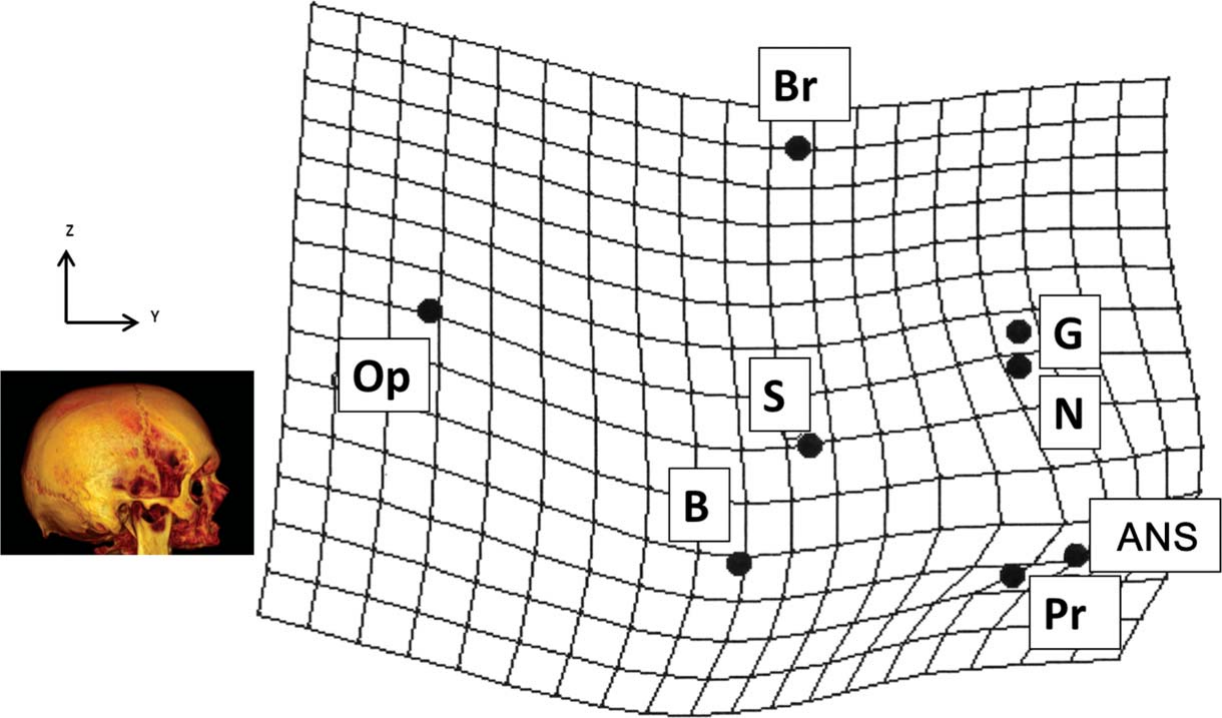
Thin-plate spline grid of the midsagittal plane of the cranium showing variations in the position of unpaired sagittal landmarks between average landmarks of ancient Graeco-Roman crania (reference shape) and average landmarks of modern crania (target shape). The grid deformations express the passage from the Graeco-Roman crania to the modern crania. Deformation is exaggerated by a factor of 2 for greater legibility. The 3D reconstructions and the axis permit a better comprehension of the orientation of the 3D graphical representation. See Table 1 for definitions of landmark abbreviations.

A thin-plate spline analysis was produced to illustrate graphically the 2D displacements of the median unpaired landmarks on a midsagittal plane (Figure 3).

The most important shape changes and landmark displacements are:

- Posterior and superior projection of the glabella and the nasion.
- Anterior projection of the anterior nasal spine.
- Upward displacement of the opisthocranion.
- Inferior displacement of the bregma, basion, and the sella.

Additionally, the CS was calculated for ancient Graeco-Roman crania and modern crania with values of 371.8 mm (standard deviation, 11.3) and 361.4 mm (standard deviation, 14.6), respectively (*P* = 0.03), indicating a statistically significant difference in size between the samples.

## Discussion

Craniometry can be used to determine different elements of the biological profile in the deceased. Methods were first developed in dry bones to objectify the subjectively different cranial features between males and females that correspond to sexual dimorphism [2]. An interest in the use of craniometry to assess geographical origin has also been demonstrated [6,7,14]. The use of geometric morphometric analysis in sciences and in particular in anthropology is not new [15]. Over time, this technique has been used for different purposes including determining ancestry, age at death, and sex. Various anatomical objects have been studied, including the cranium, mandible, and many others [16]. The appeal of this method is the possibility of quantifying shape and characterizing shape variability, permitting the objective evaluation of differences in shape and comparison with other variables while preserving all of the geometric information corresponding to the original object [17,18]. Using morphometric geometrical techniques, many studies have analyzed the development of cranial form in *Homo sapiens*, comparing modern and archaic *Homo* crania [10]. Significant differences have been found, with facial retraction and increased neurocranial globularity over time.

The comparison between dry bone measurements and measurements performed on MSCT reconstructions of identical samples has demonstrated reliability [19]. The benefits of using MSCT data are evident: no time lost in bone preparation, the potential for archiving and reuse of data allowing for increased sample size, access to subjects who are representative of the present time, and potential access to a worldwide sample. The possibility of comparing samples from different and multiple geographical origins is of major interest for every anthropologist and forensic anthropologist. Changes in craniofacial dimensions have also been studied in dry bones and more rarely with MSCT reconstructions [3,20–22].

A comparison of a North American population of the mid-nineteenth century with one from the 1970s including both Black and White subjects revealed changes in cranial dimensions [21]. That study analyzed five cranial dimensions: GOL, XCB, BBH, ZYB, and NPH. The cranial vault changes were characterized by an increased vault height (BBH) and a narrower cranial vault (XCB). Other changes were inconsistent depending on geographical origin, for example lengthening of the cranial vault (GOL). Facial changes were also found, but were inconsistent, depending on sex and geographical origin, and included a narrower face (ZYB) (for White males and females and Black females) and increased height of the face (NPH) (for White females). A study of 15 cranial dimensions also found that certain variables have changed over the past 150 years in American Black and White subjects [20]. That study confirmed that BBH had increased for both sexes and among different populations, indicating an increasing vault height. Other changes included increased values for BNL and BPL. The increased BNL corresponded with an increase in the cranial base length and a reflection of upper facial projection. For BPL, the increase corresponded with an increase in the cranial base length and a reflection of lower facial projection. However, differences were marked between Black and White subjects. XBC and ZYB tended to decrease in all groups.

Another study based on MSCT explorations performed on Italian Etruscan crania and modern Neapolitan patients also provided interesting results, with some aspects of the results contradicting those previously published by Jantz et al. [3,20,21]. In that study, as in ours, males and females were pooled together, but the number of crania included in the Etruscan study was not described in the article.

It is difficult to compare our results with those of previous studies. However, diminutions of the MAB, WFB, and BPL are in accordance with the study of Cappabianca. The NPL trend in our study is the opposite of that described by Jantz et al. [20]. However, it must be stressed that Jantz's group studied changes over 125 years whereas our study evaluated changes over 2300 years. The cranial base angle (posterior maxillary plane sella-nasion to sella-basion, SN–SB) has already been studied in different populations. The classic mean value for modern Europeans is 135.20°, (standard deviation, 5.11°) [23]. In our study, the cranial base angle was within this normal range for both ancient Graeco-Roman crania and modern crania, with values of 137.1° (standard deviation, 6°) and 138.3° (standard deviation, 7°), respectively. There were no statistically significant differences in cranial base angle between our sample populations. The classic mean value for modern Europeans for nasion-basion to nasion-anterior nasal spine (NB–NA) is 67.63°, (standard deviation, 3.70°) [23]. In our study, the NB–NA angle, representing the degree of maxillary prognathism, was not within this classic range for either ancient Graeco-Roman crania or modern crania, with values of 82.3°, (standard deviation, 4.0°) and 84.3°, (standard deviation, 4.3°), respectively. However, there were no significant differences in NB–NA angle between our sample populations.

Most of the articles evaluating secular trends in modern populations have focused on the facial cranium and have been based on 2D cephalographic studies [9,24,25]. However, the advantages of geometric morphometrics have been demonstrated, including better statistical power than distance- and angle-based methods. A comparison of nineteenth- and twentieth-century Austrian populations revealed differences, with anterior projection of the anterior nasal spine beyond the occlusion in the twentieth-century population, whereas the coronoidale was greatly displaced in an inferior direction from the pterygomaxillary fissure in its vertical coordinate only. The thin-plate spline deformation grid of the midsagittal plane landmark in our study also showed greater anterior projection of the anterior nasal spine.

An interesting study was performed on dry bones from individuals born between 1806 and 1954 in a documented skeletal collection from Lisbon [26]. The study was performed on an adult sample, differentiating males and females and using 3D geometric morphometric methods. The authors described shape changes associated with changes in size. The most important changes with increasing size were an overall decrease in cranial vault width, a more prognathic facial region, and larger and more inferior mastoid processes. Additionally, there was increased posterior cranial base flexion and inferior movement of the bregma, resulting in an overall decrease in cranial vault height. Our results partially agree with that study. We also noted changes in the posterior cranium, with a significant increase in the maximum occipital breadth. In addition, we found changes in the face, with increasing bizygomatic facial breadth (statistically non-significant) and a statistically significant increase in nasal breadth. An inferior displacement of the basion was also found in our study. However, it must be stressed that the article of Weisensee differentiated between males and females, whereas the sexes were pooled in this study. Weisensee studied changes over a 148-year period, while our study concerned data over a 2300-year period.

Deformation of the ancient crania resulting from the effects of a long period of internment is possible. We also pooled males and females in our analysis; an unequal sex distribution between ancient Graeco-Roman and modern crania could have influenced our results. The precise effects of these variables on our results are unknown and it is not possible to determine whether our findings result exclusively from cranial secular change or to other factors, including a mix of secular change and other unknown factors.

## Conclusion

The introduction of MSCT with classic craniometry and morphometric geometric techniques using landmarks and semi-landmarks opens new possibilities in the analysis and study of anthropology. The work on scanned crania based initially on exploration of dry bones (ancient Graeco-Roman crania) and living subjects (modern crania) permits an original comparison of the two samples and an evaluation of the changes in craniometric size and shape and consequently of the evolution of secular cranial changes. Unfortunately, the main limits of our work were that some factors (sex and the post-mortem effects of a long period of internment) were unknown and may have influenced our results. Changes in size consisted of a shortening of BPL, MAB, WFB, and MDH R and MDH L and elongation of the ASB and the NBL. The shape changes consisted of a global coronal enlargement of the face and cranial diameters, with the face projecting more anteriorly in the modern sample compared with the ancient Graeco-Roman sample at the anterior nasal spine, but posteriorly at the glabella and the nasion. To the best of our knowledge, MSCT-based comparisons of ancient Graeco-Roman and modern-day southern Italian crania, illustrating shape and size differences between the populations, have not previously been published.

## References

[1] Esposito V, Chiapparo S Role of anatomy in our contemporary age and the history of the Anatomy Museum of Naples. Anat Rec B New Anat. 2006;289:92–97. 16783760 10.1002/ar.b.20297

[2] Giles E, Elliot O Sex determination by discriminant function analysis of crania. Am J Phys Anthropol. 1963;21:53–68. 13947858 10.1002/ajpa.1330210108

[3] Cappabianca S, Perillo L, Esposito V, et al. A computed tomography-based comparative cephalometric analysis of the Italian craniofacial pattern through 2,700 years. Radiol Med. 2013;118:276–290. 22580801 10.1007/s11547-012-0820-z

[4] Bass WM, Missouri Archaeological Society Human osteology: a laboratory and field manual. 5th ed Columbia (MO): Missouri Archaeological Society; 2005.

[5] Ubelaker DH Human skeletal remains: excavation, analysis, interpretation. Chicago (IL): Aldine Publishing; 1978.

[6] Howells WW Criteria for selection of osteometric dimensions. Am J Phys Anthropol. 1969;30:451–457. 5791024 10.1002/ajpa.1330300317

[7] Krogman WM, MYS Iscan The human skeleton in forensic medicine. 2nd ed Springfield (IL): C.C. Thomas; 1986.

[8] Team RDC R: a language and environment for statistical computing. R Foundation for Statistical Comparative V; 2008 ISBN 3-900051-07-0 Available from: http://www.R-project.org

[9] Jonke E, Schaefer K, Freudenthaler JW, et al. A cephalometric comparison of skulls from different time periods - the Bronze Age, the 19th century and the present. Coll Antropol. 2003;27:789–801. 14746172

[10] Lieberman DE, McBratney BM, Krovitz G The evolution and development of cranial form in *Homo sapiens*. Proc Natl Acad Sci USA. 2002;99:1134–1139. 11805284 10.1073/pnas.022440799 PMC122156

[11] Braga J, Treil J Estimation of pediatric skeletal age using geometric morphometrics and three-dimensional cranial size changes. Int J Legal Med. 2007;121:439–443. 17436008 10.1007/s00414-007-0170-x

[12] Singleton M Patterns of cranial shape variation in the Papionini (Primates: Cercopithecinae). J Hum Evol. 2002;42:547–578. 11969297 10.1006/jhev.2001.0539

[13] von Cramon-Taubadel N, Frazier BC, Lahr MM The problem of assessing landmark error in geometric morphometrics: theory, methods, and modifications. Am J Phys Anthropol. 2007;134:24–35. 17503448 10.1002/ajpa.20616

[14] Kallenberger L, Pilbrow V Using CRANID to test the population affinity of known crania. J Anat. 2012;221:459–464. 22924771 10.1111/j.1469-7580.2012.01558.x PMC3482354

[15] Slice DE Modern morphometrics in physical anthropology. New York (NY): Kluwer Academic; 2005.

[16] Bigoni L, Veleminska J, Bruzek J Three-dimensional geometric morphometric analysis of cranio-facial sexual dimorphism in a Central European sample of known sex. Homo. 2010;61:16–32. 20152969 10.1016/j.jchb.2009.09.004

[17] Zelditch M Geometric morphometrics for biologists: a primer. Amsterdam: Elsevier Academic Press; 2004.

[18] Bookstein FL Morphometric tools for landmark data: geometry and biology. Cambridge: Cambridge University Press; 1991.

[19] Verhoff MA, Ramsthaler F, Krahahn J, et al. Digital forensic osteology–possibilities in cooperation with the Virtopsy project. Forensic Sci Int. 2008;174:152–156. 17451898 10.1016/j.forsciint.2007.03.017

[20] Jantz RL Cranial change in Americans: 1850–1975. J Forensic Sci. 2001;46:784–787. 11451056

[21] Jantz RL, Meadows Jantz L Secular change in craniofacial morphology. Am J Hum Biol. 2000;12:327–338. 11534023 10.1002/(SICI)1520-6300(200005/06)12:3<327::AID-AJHB3>3.0.CO;2-1

[22] Jantz RL, Owsley DW Variation among early North American crania. Am J Phys Anthropol. 2001;114:146–655. 11169904 10.1002/1096-8644(200102)114:2<146::AID-AJPA1014>3.0.CO;2-E

[23] Kuroe K, Rosas A, Molleson T Variation in the cranial base orientation and facial skeleton in dry skulls sampled from three major populations. Eur J Orthod. 2004;26:201–207. 15130044 10.1093/ejo/26.2.201

[24] Jonke E, Prossinger H, Bookstein FL, et al. Secular trends in the facial skull from the 19th century to the present, analyzed with geometric morphometrics. Am J Orthod Dentofacial Orthop. 2007;132:63–70. 17628252 10.1016/j.ajodo.2005.08.040

[25] Jonke E, Prossinger H, Bookstein FL, et al. Secular trends in the European male facial skull from the migration period to the present: a cephalometric study. Eur J Orthod. 2008;30:614–620. 19054815 10.1093/ejo/cjn065

[26] Weisensee KE, Jantz RL Secular changes in craniofacial morphology of the Portuguese using geometric morphometrics. Am J Phys Anthropol. 2011;145:548–559. 21541933 10.1002/ajpa.21531

